# Time to publication for NIHR HTA programme-funded studies

**DOI:** 10.1186/1745-6215-14-S1-P69

**Published:** 2013-11-29

**Authors:** Fay Chinnery, Amanda Young, Jennie Goodman, Ruairidh Milne, Martin Ashton-Key

**Affiliations:** 1 National Institute for Health Research, Evaluation, Trials and Studies Coordinating Centre, Southampton, UK; 2 Wessex Institute, Southampton, UK

## Objective

Previous work has shown that 98% of NIHR Health Technology Assessment (HTA) Programme projects publish in *Health Technology Assessment* (the peer reviewed journal for the NIHR HTA Programme). 68% of clinical trials funded by the US NIH publish, with 46% publishing within 30 months of trial completion. This study aimed to assess the time-to-publication of HTA Programme primary research (trials, diagnostic studies, surveys and modelling projects) and compare this with other funding bodies.

## Study design and setting

A retrospective cohort study of HTA Programme primary research, completing between 1993 and 2011, publishing in *Health Technology Assessment* and any associated publications in other peer-reviewed journals. Time-to-publication was determined by calculating the number of months from when the study concluded (end of follow-up) to the online publication date in *Health Technology Assessment* or external journal.

## Results

92.3% of HTA Programme primary research projects in this cohort (n=155) published and the median time to publication was 22 months. 88.4% of primary research studies published in *Health Technology Assessment*, with 62.6% publishing in another peer-reviewed journal. 93.7% of HTA Programme trials (n=126) published, 67.5% by 30 months and the median time to publication was 23 months.

## Conclusion

HTA Programme research publishes promptly and the importance of *Health Technology Assessment* was highlighted, as publication rate for primary research was considerably higher in this than for other peer-reviewed journals. HTA Programme trials also have a considerably higher publication rate and publish more promptly than trials funded by the US NIH and those sponsored by industry.

